# Evaluating the therapeutic role of selected active compounds in *Plumula Nelumbinis* on pulmonary hypertension *via* network pharmacology and experimental analysis

**DOI:** 10.3389/fphar.2022.977921

**Published:** 2022-08-17

**Authors:** Xinghua Xiao, Fangmei Luo, Minyi Fu, Yueping Jiang, Shao Liu, Bin Liu

**Affiliations:** ^1^ Department of Pharmacy, Xiangya Hospital, Central South University, Changsha, China; ^2^ Institute for Rational and Safe Medication Practices, National Clinical Research Center for Geriatric Disorders, Xiangya Hospital, Central South University, Changsha, China; ^3^ The Hunan Institute of Pharmacy Practice and Clinical Research, Xiangya Hospital, Central South University, Changsha, China; ^4^ Department of Pharmacy, Hunan Children’s Hospital, Changsha, China

**Keywords:** pulmonary hypertension, network pharmacology, *Plumula Nelumbinis*, alkaloids, PASMCs proliferation, PASMCs migration

## Abstract

Excessive proliferation and migration of pulmonary artery smooth muscle cells (PASMCs) are critical factors leading to vascular remodeling in pulmonary hypertension (PH). This study aimed to explore the effect and potential mechanism of *Plumula Nelumbinis* on PH by using network pharmacology and experimental analysis. Network pharmacology and molecular docking results indicated that the potential active components of *Plumula Nelumbinis* against PH were mainly alkaloid compounds, including neferine, liensinine, and isoliensinine. Subsequently, by constructing a Su5416 plus hypoxia (SuHx)-induced PH rat model, we found that the total alkaloids of *Plumula Nelumbinis* (TAPN) can reduce the right ventricular systolic pressure, delay the process of pulmonary vascular and right ventricular remodeling, and improve the right heart function in PH rats. In addition, TAPN can effectively reverse the upregulation of collagen1, collagen3, MMP2, MMP9, PCNA, PIM1, and p-SRC protein expression in lung tissue of PH rats. Finally, by constructing a hypoxia-induced PASMCs proliferation and migration model, we further found that TAPN, neferine, liensinine, and isoliensinine could inhibit the proliferation and migration of PASMCs induced by hypoxia; reverse the upregulation of collagen1, collagen3, MMP2, MMP9, PCNA, PIM1 and p-SRC protein expression in PASMCs. Based on these observations, we conclude that the alkaloid compounds extracted from *Plumula Nelumbinis* (such as neferine, liensinine, and isoliensinine) can inhibit the abnormal proliferation and migration of PASMCs by regulating the expression of p-SRC and PIM1, thereby delaying the progression of PH.

## Introduction

Pulmonary hypertension (PH) is a malignant cardiopulmonary vascular disease characterized by persistently elevated pulmonary arterial pressure (Poch and Mandel, 2021; Walter, 2021). Pulmonary vascular remodeling is the most basic pathological feature of PH, involving processes such as the excessive proliferation and migration of pulmonary artery smooth muscle cells (PASMCs), endothelial dysfunction, and extracellular matrix deposition (Shimoda, 2020). Currently approved drugs for the treatment of PH mainly target three molecular pathways related to the pathogenesis of PH: prostacyclins and prostacyclin-receptor agonists (such as epoprostenol, iloprost, selexipag, and treprostinil), endothelin receptor antagonists (such as ambrisentan, bosentan, and macitentan), and nitric oxide pathway agents (such as sildenafil and tadalafil) (Poch and Mandel, 2021). However, the above existing drug treatment usually only improves symptoms and cannot achieve the purpose of a radical cure. Therefore, finding more safe and effective drugs to prevent and treat PH is urgent.

Network pharmacology is a useful bioinformatics tool to reveal the complex biological network relationship between drugs, targets, and diseases by using high-throughput screening, network visualization, and analysis techniques (Nogales et al., 2021). In recent years, with the rise of network pharmacology, systematic research on the underlying mechanisms of traditional Chinese medicine (TCM) has been widely carried out, providing a foundation for the modernization of TCM (An et al., 2021). Accumulating evidence suggests that active ingredients derived from TCM, such as magnolol (Fu et al., 2021), magnesium lithospermate B (Li et al., 2019b), and resveratrol (Mirhadi et al., 2021), could delay the process of pulmonary vascular and right ventricular remodeling during PH, which brings a promising future for the prevention and treatment of PH.

*Plumula Nelumbinis*, a TCM derived from the dried young leaves and radicles of the mature seeds of the water lily plant *Nelumbo nucifera Gaertn*, could be used for medicine and food. It has multiple pharmacological activities such as cardiovascular protection, anti-oxidation, anti-inflammatory, and anti-tumor (Chen et al., 2021). Recent studies have shown that alkaloids from *Plumula Nelumbinis* could attenuate vascular remodeling in spontaneously hypertensive rats (Li Q et al., 2019; Wicha et al., 2020). However, its effect on vascular remodeling in PH is still unclear.

**FIGURE 1:**
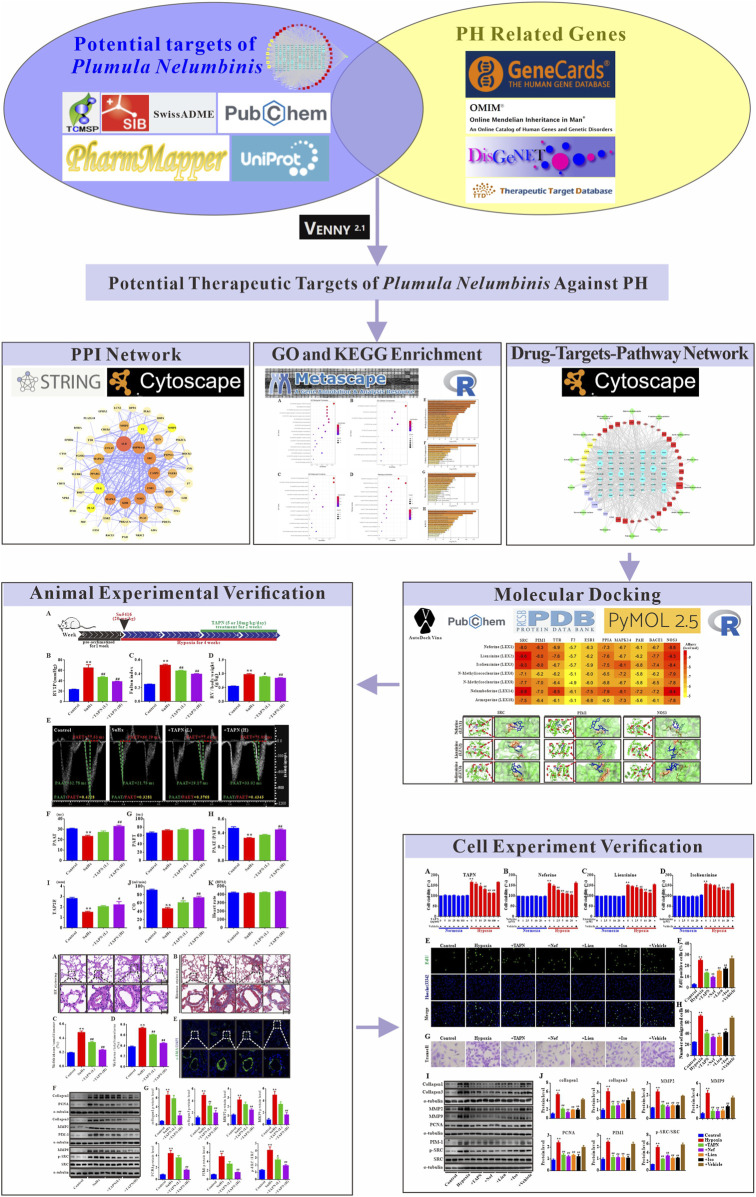
The network pharmacology workflow of *Plumula Nelumbinis* against pulmonary hypertension.

In this study, we first screened out the main active components and targets of *Plumula Nelumbinis* against PH through a network pharmacology strategy and then performed GO function and KEGG pathway enrichment analysis. Next, the interaction between the core compounds and targets was simulated by molecular docking. Finally, by establishing a Su5416 plus hypoxia (SuHx) induced PH rats model and a hypoxia-induced PASMCs proliferation model, the prediction results were experimentally verified from *in vivo* and *in vitro* levels. The overall design of this study is shown in Figure 1. The results of this study can further clarify the mechanism of *Plumula Nelumbinis* in treating PH and provide a basis for developing novel anti-PH drugs.

## Materials and methods

### Identification of the main active ingredient of *Plumula Nelumbinis*

The active ingredients of *Plumula Nelumbinis* were obtained from the TCMSP database (Traditional Chinese Medicine Systems Pharmacology and Analysis Platform, https://old.tcmsp-e.com/tcmsp.php) (Ru et al., 2014) and related literature (Jiang et al., 2018b; Chen et al., 2021) by using search terms such as “*Plumula Nelumbinis*” or “*Hindu Lotus Plumule*”. The chemical information (such as PubChem CID, 3D conformer, and canonical SMILES) and pharmacokinetic parameters of these active ingredients were collected from the PubChem (https://pubchem.ncbi.nlm.nih.gov/) and SwissADME (http://www.swissadme.ch/) database. The main active ingredients of *Plumula Nelumbinis* were screened according to the following conditions as described in the previous study (Daina et al., 2017): 1) The gastrointestinal absorption coefficient was set to High, which indicates that these compounds have good oral bioavailability and could be effectively absorbed; 2) The flexibility was evaluated by rotatable bonds: rotatable bonds ≤10; 3) The polarity was evaluated by topological polar surface area (TPSA): 20 Å^2^≤ TPSA ≤140 Å^2^; 4) The drug-likeness ability was evaluated by five different rule-based filters (Lipinski, Chose, Veber, Egan, and Muegge filter) and the bioavailability score: the bioavailability score ≥10%. The potential targets of the main active ingredients of *Plumula Nelumbinis* were predicted through the PharmMapper database (http://www.lilab-ecust.cn/pharmmapper/submitfile.html, Normalized Fit Score ≥0.70) (Wang et al., 2017). The potential targets were identified and normalized through the UniProt database (https://www.uniprot.org/).

### Identification of PH-Related targets

The PH-related targets were obtained from GeneCards (https://www.genecards.org/), Online Mendelian Inheritance in Man (OMIM, https://omim.org/) (Amberger et al., 2019), DisGeNET (https://www.disgenet.org/) (Pinero et al., 2021), and Therapeutic Target Database (TTD, http://db.idrblab.net/ttd/) (Zhou et al., 2021) by using search terms such as “pulmonary hypertension” or “pulmonary arterial hypertension” (accessed on 21 October 2021). The above targets were summarized, de-duplicated, and then identified as PH-related targets.

### Construction of drug-target-disease and protein-protein interaction network

The intersection between the potential targets of *Plumula Nelumbinis* and the PH-related targets was obtained by the VENNY online tool (https://bioinfogp.cnb.csic.es/tools/venny/index.html). The overlapping targets were considered as the potential therapeutic targets of *Plumula Nelumbinis* against PH. The protein interaction relationships of the overlapped targets were acquired through the STRING database (https://cn.string-db.org/, Version 11.5) (Szklarczyk et al., 2017). In brief, the overlapped targets were uploaded to the STRING database, and the species was limited to “*Homo sapiens*”. Finally, the obtained PPI network information of the intersection targets was led to the Cytoscape software for visual analysis (https://cytoscape.org/, Version 3.8.2) (Shannon et al., 2003).

### GO and KEGG pathway enrichment analysis

The Metascape online tool (https://metascape.org/) was applied for GO and KEGG enrichment analysis (Zhou et al., 2019). Briefly, the potential therapeutic targets of *Plumula Nelumbinis* against PH were uploaded to the Metascape platform. The filter thresholds of GO function and KEGG pathway analysis were set as the following conditions: 1) the species was set as “*Homo sapiens*”; 2) the minimum overlap value was set to 3; 3) the *P* cutoff value was set to 0.01; 4) the minimum enrichment value was set to 1.5. The GO and KEGG enrichment analysis results were visualized through the ggplot2 package (Version 3.3.5) of the R language (Version 4.0.2) (Wickham et al., 2016).

### Construction of the drug-target-pathway network

The main component-target-pathway enrichment network was visualized using the Cytoscape software (Shannon et al., 2003). The supporting tool “Network Analyzer” of Cytoscape software was used to obtain the network topology parameters (such as degree, betweenness, and closeness) between the effective ingredients and targets. The top-ranked compounds and targets based on degree, betweenness, and closeness were defined as the core compounds and targets. These core compounds and targets were subsequently prepared for molecular docking.

### Molecular docking

The binding situation of core compounds and targets was analyzed using AutoDock Vina software (https://vina.scripps.edu/, Version 1.1.2). Firstly, the three-dimensional crystal structures of the top 10 core target proteins were obtained from the Protein Data Bank (PDB, https://www.pdbus.org/) (Burley et al., 2021). The PDB ID of these target proteins are as follows: proto-oncogene tyrosine-protein kinase Src (SRC, PDB ID: 2SRC); serine/threonine-protein kinase PIM-1 (PIM1, PDBID:1XWS); transthyretin (TTR, PDB ID:1BZE); prothrombin (F2, PDB ID:2CN0); estrogen receptor-α (ESR1, PDB ID:5FQV); cyclophilin A (PPIA, PDB ID: 5LUD); mitogen-activated protein kinase 14 (MAPK14, PDB ID:2FST); phenylalanine-4-hydroxylase (PAH, PDB ID:5FII); β-secretase 1 (BACE1, PDB ID: 1TQF); endothelial nitric oxide synthase (NOS3, PDB ID:1M9M). Secondly, the 3D structures of the core compounds were obtained from the PubChem database (https://pubchem.ncbi.nlm.nih.gov/) and then were converted into PDB file format by using OPEN Babel software (http://openbabel.org/, Version 2.4.1) (O'Boyle et al., 2011). Finally, the molecular docking of core compounds and targets was performed using AutoDock Vina software (Trott and Olson, 2010). The binding energy volcano map was drawn through the pheatmap package (Version 1.0.12) of the R language. At the same time, the molecular docking results were visualized using the PyMol software (Version 2.2.0). The binding affinity value <0 kcal/mol indicates that the compounds effectively bind to the targets.

### Chemicals and reagents

The separation, extraction, and identification of the total alkaloids from *Plumula Nelumbinis* (TAPN), neferine, liensinine, and isoliensinine were performed as described in our previous study (Liu et al., 2009; Jiang et al., 2018a; Chen et al., 2020). Dimethyl sulfoxide (DMSO) was purchased from Sigma-Aldrich (C6164, Darmstadt, Germany). High-glucose Dulbecco’s modified Eagle’s medium (DMEM) and fetal bovine serum (FBS) were purchased from Gibco (Thermo Fisher Scientific). Primary antibodies against collagen1, collagen3, MMP2, MMP9, PCNA, PIM1, p-SRC, and SRC were purchased from Beyotime (Shanghai, China). α-Tubulin was purchased from Santa Cruz Biotechnology (CA, United States).

### Establishment of the SU5416/hypoxia-induced PH rat model

To validate the predicted results of network pharmacology and molecular docking, we further evaluated the therapeutic effect of TAPN on PH from the *in vivo* level. The initial doses of TAPN *in vivo* (5 mg/kg/d and 10 mg/kg/d) were determined based on previous studies (Li Q et al., 2019). SuHx-induced PH rat model was constructed as described in the previous study (Legchenko et al., 2018). SD rats (∼160 g) were randomly divided into the following 4 groups (n = 12 per group): 1) the normoxia group, rats were given the same volume of vehicle (a mixture with 5% DMSO, 30% PEG300, 5% Tween-80, and 60% saline) and then placed in the normoxic environment for 4 weeks; 2) the SuHx group, rats were given a single subcutaneous injection of Su5416 (20 mg/kg, S832952, Macklin, Shanghai, China) and then placed immediately into a hypoxic chamber (10% O_2_) for 4 weeks; 3) the SuHx plus TAPN (L) group (low dose, 5 mg/kg/d); 4) and the SuHx plus TAPN (H) group (high dose, 10 mg/kg/d). After the injection of Su5416 plus hypoxia for 2 weeks, the rats were administered with TAPN at 5 or 10 mg/kg (i.g.,) once a day for 2 weeks. After 4 weeks of SuHx treatment, the cardiac function of rats was detected by Doppler echocardiography. The right ventricular systolic pressure (RVSP) in rats was measured by the right heart catheterization. The Fulton index (RV/LV + IVS) and the ratio of RV weight to body weight were used to assess the degree of right ventricular remodeling. The lung tissue of rats was collected for subsequent molecular biology and morphological analysis.

### Morphological analysis for lung tissue

Hematoxylin-eosin (HE), Masson, and immunofluorescence staining were used to evaluate the morphological changes in lung tissues. The detailed procedures for morphological analysis were performed as described in our previous studies (Wang et al., 2019; Xiao et al., 2022). The proportion of vessel wall thickness (ratio of wall thickness to vessel diameter, WT%) and the proportion of vessel wall area (ratio of wall area to total vessel area, WA%) were used to measure the severity of pulmonary vascular remodeling. In addition, α-SMA immunofluorescence staining was used to assess the degree of pulmonary vascular media thickening.

### Western blotting

The lung tissues or PASMCs were homogenized in RIPA lysis buffer (Beyotime, Shanghai, China) with a protease and phosphatase inhibitor cocktail (Beyotime, Shanghai, China). Samples containing 20–40 μg of protein were separated by 10% SDS-PAGE gel and then transferred to polyvinylidene fluoride (PVDF) membranes (G.E. Healthcare, Germany). After blocking with 5% skim milk or 5% BSA, the PVDF membranes were incubated overnight on a shaker at 4°C with the following primary antibodies: collagen1 (AF6524, 1:1,000, Beyotime), collagen3 (AF6531, 1:1,000, Beyotime), MMP2 (AF0234, 1:1,000, Beyotime), MMP9(AF5234, 1:1,000, Beyotime), PCNA (AF0261, 1:1,000, Beyotime), SRC (AF1831, 1:1,000, Beyotime), p-SRC (AF5923, 1:1,000, Beyotime), PIM1 (AF1807, 1:1,000, Beyotime), eNOS (AF6792, 1:1,000, Beyotime), and α-tubulin (sc-5286, 1:500, Santa Cruz). After incubation with horseradish peroxidase (HRP)-linked secondary antibody (Beyotime, Shanghai, China), the protein bands were imaged through Molecular Imager ChemiDoc XRS System (Bio-Rad, Philadelphia, USA). Densitometric quantification was performed by ImageJ (NIH, USA). The α-tubulin served as a loading control.

### Cell experiments

To further clarify the underlying mechanism of TAPN against PH, we constructed a hypoxia-induced proliferation and migration model of PASMCs. The initial doses of TAPN, neferine, liensinine, and isoliensinine were determined based on previous studies (Jun et al., 2016). The isolation of PASMCs from pulmonary arteries of rats and the hypoxia-induced PASMCs proliferation model were performed as described in our previous study (Li et al., 2019a; Xiao et al., 2022). PASMCs at passages 3 to 6 were used for cell identification and the subsequent experiments. In order to evaluate the effect of the screened main active components of *Plumula Nelumbinis* on the proliferation of PASMCs induced by hypoxia, the cell experiments were grouped as follows: 1) the normoxia group, PASMCs were cultured in a normoxic incubator (21% O_2_) for 48 h; 2) the hypoxia group, PASMCs were cultured in a hypoxia incubator (3% O_2_) for 48 h; 3) the hypoxia plus drug intervention group, PASMCs were pretreated with different doses of drugs for 1 h and then treated with hypoxia for 48 h, the initial drug doses for neferine, liensinine, and isoliensinine were set at 1, 2.5, 5, 10, and 20 μM, respectively, while the initial doses of TAPN were 5, 10, 25, 50 and 100 μg/ml, respectively. 4) the hypoxia plus vehicle group, PASMCs were pretreated with an equal volume of vehicle (DMSO, volume ≤1/1,000 of the total medium) for 1 h and then treated with hypoxia for 48 h. At the end of the experiments, the PASMCs were collected for cell proliferation detection and molecular analysis.

### Cell proliferation assay

Cell proliferation was detected by CCK-8 Assay Kit (C6005M, UElandy, Suzhou, China) and EdU Assay Kit (C6017L, UElandy, Suzhou, China) according to the protocol provided by the manufacturer. For the CCK-8 assay, PASMCs were seeded at 96-well plates and then starved with a high-glucose DMEM culture medium containing 1% fetal bovine serum (FBS) for 24 h. After drug and hypoxia intervention, the CCK-8 solution (10 μL) was added to each well and then incubated in the dark for 2 h in the 37°C cell incubator. The optical density (OD) value was detected at 450 nm with SpectraMax 190 (Molecular Devices, California, USA).

For the EdU array, PASMCs were inoculated into the 96-well plates and synchronized for 24 h when cell density reached 50%. After drug and hypoxia intervention, the EdU solution (50 μM) was added to each well and then incubated for 2 h in the 37°C cell incubator. After fixation with 4% (w/v) paraformaldehyde and treatment with 0.5% Triton X-100 for 20 min, EdU working solution and Hoechst 33,342 were added for staining, respectively. The number of EdU positive cells was observed under a fluorescence microscope, and the positive cells in each group were counted and analyzed by ImageJ (Version 1.46).

### Cell migration assay

Cell migration ability was measured by Transwell assay as described in our previous studies (Wang et al., 2019; Xiao et al., 2022). In brief, 1 × 10^5^ PASMCs were seeded into the upper chamber of the 24-well Transwell plate (3,422, Corning, NY, USA). After serum starvation treatment for 12 h, the DMEM culture medium containing 20% FBS was added into the lower chamber, and then the cells were treated with hypoxia and drugs for 48 h. Cells attached to the upper surface were removed with a cotton swab, while the migrated cells on the lower surface were incubated in 4% paraformaldehyde and 0.1% crystal violet solution (C0121, Beyotime, Shanghai, China). The number of migrated cells was observed and counted under an optical microscope.

### Statistical analysis

All quantitative data were presented as the means ± standard error of mean (S.E.M) and analyzed using SPSS 20.0 software (SPSS, Chicago, United States). One-way analysis of variance (ANOVA) was used to compare the means among different groups. *p* ≤ 0.05 was considered statistically significant.

## Results

### Screening of main active compounds from *Plumula Nelumbinis*

More than 100 compounds have been isolated and identified from the *Plumula Nelumbinis*, including alkaloids (such as neferine, liensinine, and isoliensinine), flavonoids (such as quercetin, rutin, and kaempferol), volatile oil, and polysaccharides. A total of 92 possible active compounds were initially screened by matching with information from the PubChem database. Then the pharmacokinetic parameters of those compounds were obtained from the SwissADME database. Next, we conducted a secondary screening of those compounds based on the gastrointestinal absorption rate, flexibility, polarity, and drug-likeness. A total of 46 main active ingredients of *Plumula Nelumbinis* were finally obtained, including 30 alkaloids, 7 flavonoids, and 9 other compounds (volatile oil or polysaccharides). The above 46 compounds are numbered LZX1-LZX46; relevant information is shown in Table 1.

**TABLE 1:** The main active ingredients of *Plumula Nelumbinis*.

Number	Pubchem name	PubChem ID	Class
LZX1	Neferine	159654	Alkaloids
LZX2	Liensinine	160644	Alkaloids
LZX3	Isoliensinine	5274591	Alkaloids
LZX4	Higenamine	114840	Alkaloids
LZX5	(S)-isococlaurine	138319297	Alkaloids
LZX6	(+)-N-Methylisococlaurine	21817819	Alkaloids
LZX7	Coclaurine	160487	Alkaloids
LZX8	(R)-N-Methylcoclaurine	440595	Alkaloids
LZX9	Lotusine	5274587	Alkaloids
LZX10	N-Nornuciferine	12313579	Alkaloids
LZX11	Nuciferine	10146	Alkaloids
LZX12	O-Nornuciferine	197017	Alkaloids
LZX13	Nornuciferidine	183520	Alkaloids
LZX14	Nelumboferine	76046684	Alkaloids
LZX15	Dauricine	73400	Alkaloids
LZX16	Pronuciferine	200480	Alkaloids
LZX17	Berberine	2353	Alkaloids
LZX18	(-)-Armepavine	442169	Alkaloids
LZX19	Lysicamine	122691	Alkaloids
LZX20	Anonaine	160597	Alkaloids
LZX21	Roemerine	119204	Alkaloids
LZX22	Liriodenine	10144	Alkaloids
LZX23	Thalifoline	89048	Alkaloids
LZX24	N-Methylcorydaldine	303906	Alkaloids
LZX25	2-Methyl-1H-indole-3-carbaldehyde	73166	Alkaloids
LZX26	(R)-Norcoclaurine	440988	Alkaloids
LZX27	1alpha-(4-Methoxybenzyl)-2-methyl-6-methoxy-1,2,3,4-tetrahydroisoquinoline	132579667	Alkaloids
LZX28	N-Methylisosalsoline	40091	Alkaloids
LZX29	O-Methylarmepavine	821338	Alkaloids
LZX30	6,7-Dimethoxy-2-methyl-1,2,3,4-tetrahydroisoquinoline	27694	Alkaloids
LZX31	Quercetin	5280343	Flavonoids
LZX32	Isorhamnetin	5281654	Flavonoids
LZX33	Kaempferol	5280863	Flavonoids
LZX34	Diosmetin	5281612	Flavonoids
LZX35	Naringenin	932	Flavonoids
LZX36	Luteolin	5280445	Flavonoids
LZX37	Tricetin	5281701	Flavonoids
LZX38	5-Hydroxymethylfurfural	237332	Others
LZX39	Linalool	6549	Others
LZX40	(1,7,7-Trimethyl-2-bicyclo [2.2.1]heptanyl) 3-phenylprop-2-enoate	583021	Others
LZX41	Gallic acid	370	Others
LZX42	Dibutyl phthalate	3026	Others
LZX43	Gamabufotalin	259803	Others
LZX44	Loureirin A	5319081	Others
LZX45	Methyl 4-hydroxycinnamate	5319562	Others
LZX46	Propanethial S-oxide	441491	Others

### Identification of potential targets of *Plumula Nelumbinis* against PH

**FIGURE 2:**
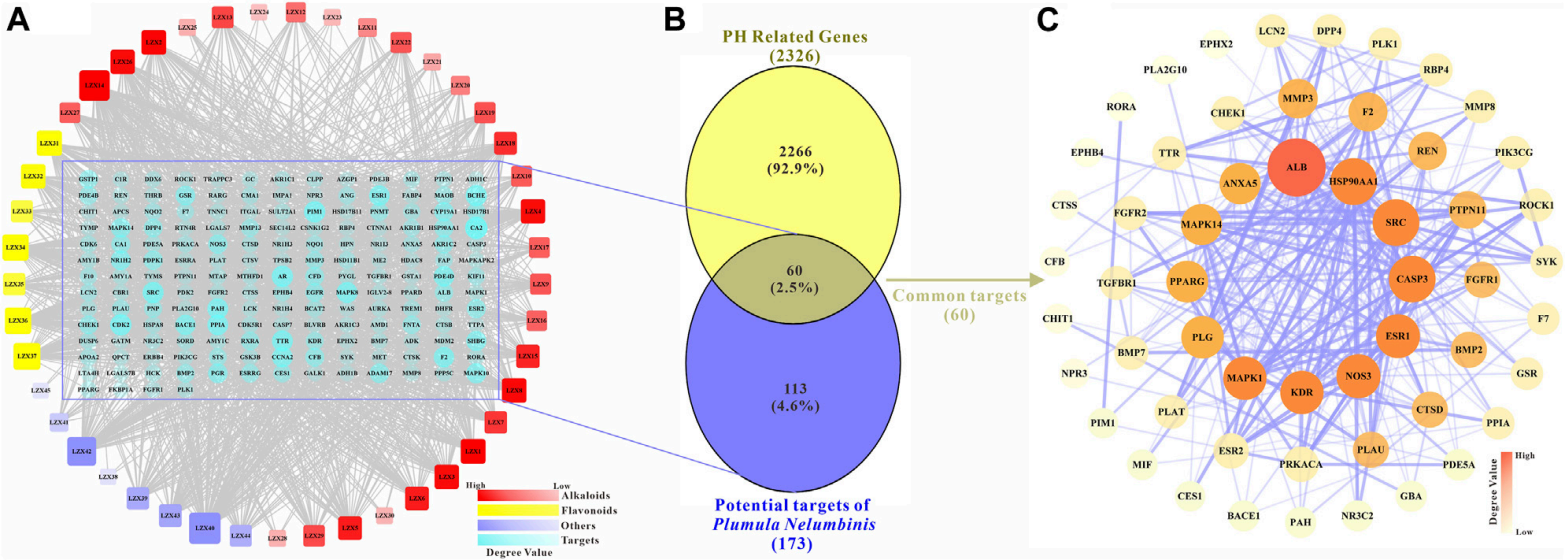
**(A)** The main active components and potential targets of *Plumula Nelumbinis*. The circles represent targets (173 in total). The squares represent the active compounds from *Plumula Nelumbinis* (45 in total), including 30 alkaloids (marked in red), 7 flavonoids (marked in yellow), and 8 other compounds (volatile oils or polysaccharides, marked in purple). **(B)** Venn diagram of potential targets of the main active components of *Plumula Nelumbinis* against PH. The intersection of the potential targets of *Plumula Nelumbinis* and PH-related targets was obtained by using the VENNY 2.1 online tool, which was identified as the potential targets of *Plumula Nelumbinis* against PH **(C)** PPI network diagram of potential targets of *Plumula Nelumbinis* against PH.

The potential targets of the main active ingredients were obtained from the PharmMapper database. LZX46 (Propanethial S-oxide) was excluded because the predicted target of LZX46 was not available in the PharmMapper database. A total of 173 potential targets for the other 45 compounds were obtained after deduplication. The drug-target network is shown in Figure 2A.

After summarizing and de-duplicating the targets, a total of 2326 PH-related targets were collected from GeneCards, OMIM, DisGeNET, and TTD databases. As shown in Figure 2B, 60 common targets were obtained after the intersection of *Plumula Nelumbinis*-related and PH-related targets. These common targets were identified as potential therapeutic targets of *Plumula Nelumbinis* against PH, and the interaction relationship between these targets is shown in Figure 2C.

### GO and KEGG enrichment analysis of common targets

**FIGURE 3:**
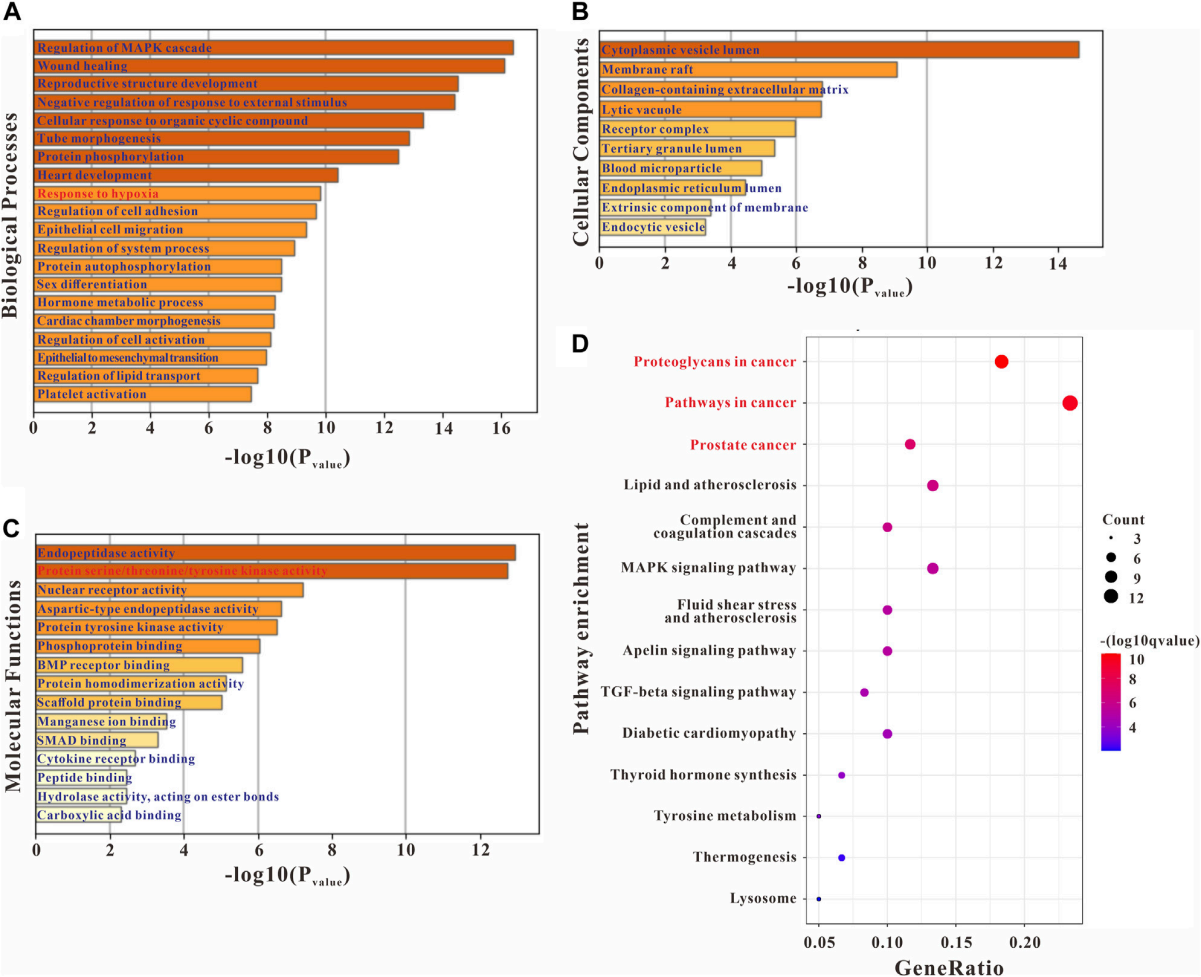
GO and KEGG enrichment analysis of the potential targets of *Plumula Nelumbinis* against PH. **(A)** GO biological processes analysis **(B)** GO molecular functions analysis **(C)** GO cellular components analysis **(D)** KEGG pathway enrichment analysis.

GO enrichment analyses were conducted to confirm the intersection targets’ function. GO function analysis results showed that the anti-PH effect of *Plumula Nelumbinis* might be closely related to a variety of biological processes (BP), including regulation of MAPK cascade (GO:0043408), wound healing (GO:0042060), reproductive structure development (GO:0048608), negative regulation of response to external stimulus (GO:0032102), cellular response to organic cyclic compound (GO:0071407), tube morphogenesis (GO:0035239), and protein phosphorylation (GO:0006468) (Figure 3A). GO cellular components (CC) analysis terms mainly include cytoplasmic vesicle lumen (GO:0060205), membrane raft (GO:0045121), collagen-containing extracellular matrix (GO:0062023), lytic vacuole (GO:0000323), and receptor complex (GO:0043235) (Figure 3B). GO molecular function (MF) analysis terms mainly include endopeptidase activity (GO:0004175) and protein serine/threonine/tyrosine kinase activity (GO:0004712) (Figure 3C). KEGG pathway enrichment analysis indicated that the anti-PH effect of *Plumula Nelumbinis* might be achieved by regulating the following pathways: proteoglycans in cancer (hsa05205) and pathways in cancer (hsa05200) (Figure 3D).

### Identification of core compounds and targets of *Plumula Nelumbinis*

**FIGURE 4:**
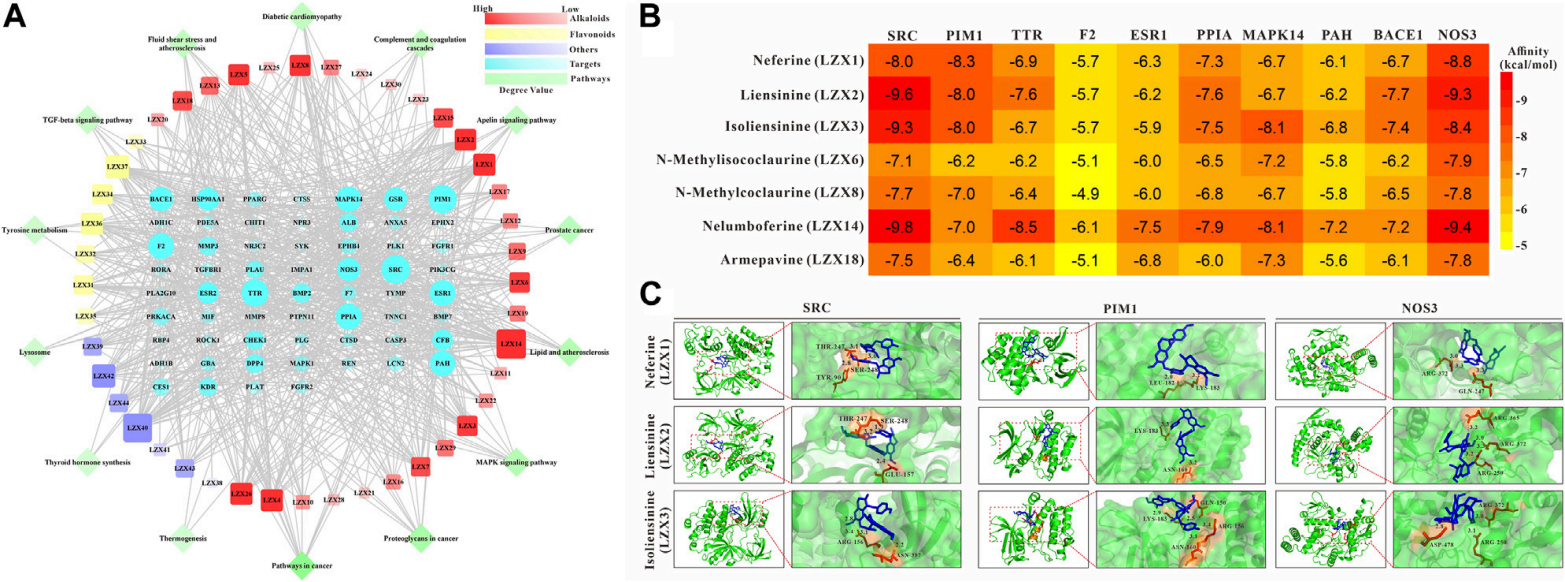
**(A)** Compounds-targets-pathways network analysis diagram **(B)** The interaction between 7 candidate core compounds and 10 core targets was evaluated by AutoDock Vina. **(C)** The molecular docking pattern between the core compounds and targets.

The compounds-targets-pathways network was constructed to clarify the core compounds and corresponding targets of *Plumula Nelumbinis* in PH treatment (Figure 4A). We first screened out nodes with degrees ≥25 in the compound-target-pathway network as core targets, namely SRC, PIM1, TTR, F2, ESR1, PPIA, MAPK14, PAH, BACE1, and NOS3, respectively. Then the top 7 candidate core compounds were screened out according to the network topological parameters with the core targets, namely LZX1 (Neferine), LZX2 (Liensinine), LZX3 (Isoliensinine), LZX6 (N-Methylisococlaurine), LZX8 (N-Methylcoclaurine), LZX14 (Nelumboferine), and LZX18 (Armepavine).

In order to determine the possible binding interaction between core compounds and targets, molecular docking analysis was performed using AutoDock Vina. The molecular docking results showed that the average binding energy between the core compounds and targets is -7.03 kcal/mol, indicating an effective binding between the compounds and targets. As we all know, the lower the binding energy score, the stronger the compound’s ability to bind to the targets. By setting the binding energy threshold to −8.00 kcal/mol, we finally obtained three compounds (LZX1, LZX2, and LZX3) and three targets (SRC, PIM1, and NOS3) for subsequent experiments verification (Figure 4B). The molecular docking pattern between the core compounds and targets is shown in Figure 4C.

### Effects of TAPN on hemodynamics and right ventricular function in SuHx-induced PH rats

**FIGURE 5:**
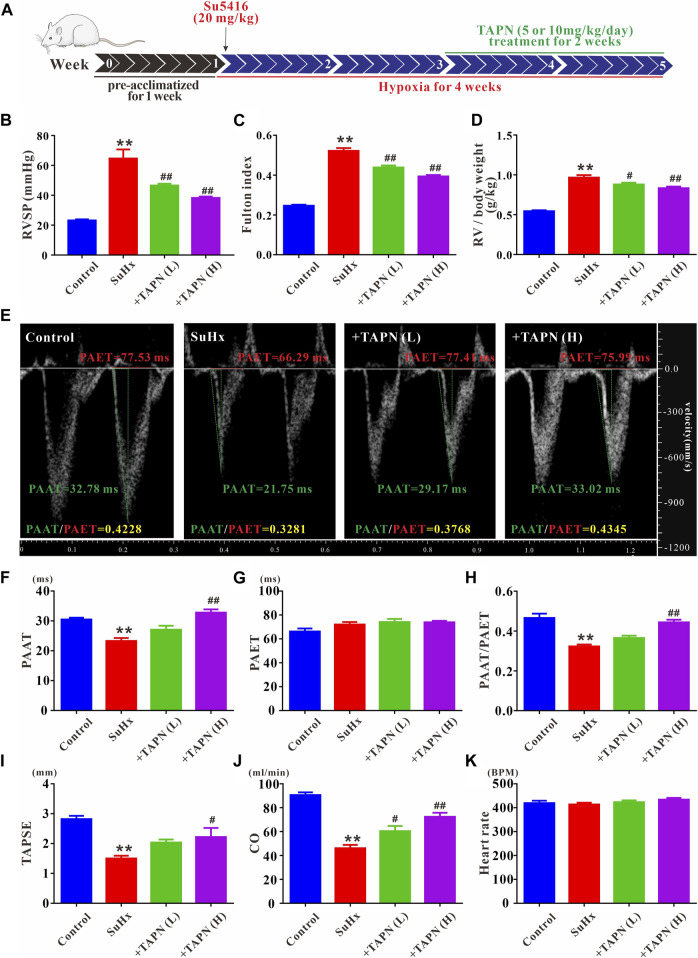
TAPN inhibits right ventricular remodeling and right heart dysfunction in SuHx rats. **(A)** Design diagram of *in vivo* drug administration experiment in SD rats **(B)** RVSP in each group **(C)** Fulton index: RV/LV + IVS **(D)** The ratio of RV weight to body weight **(E)** Representative images of pulsed Doppler from pulmonary artery flow tract recorded in parasternal long axis, the pulmonary artery acceleration time (PAAT, green horizontal line), the pulmonary artery ejection time (PAET, red horizontal line) **(F)** The value of PAAT in each group **(G)** The value of PAET in each group **(H)** The ratio of PAAT to PAET **(I)** The value of tricuspid annular plane systolic excursion (TAPSE) **(J)** Cardiac output (CO) **(K)** Heart rate. n = 8–12 per group, Data are expressed as mean ± SEM. SuHx: the Su5416 + hypoxia group; +TAPN (L): SuHx + TAPN low dose group (5 mg/kg/d); +TAPN **(H)**: SuHx + TAPN high dose group (10 mg/kg/d); ^**^ *p* < 0.01 vs. Control; ^#^ *p* < 0.05, ^##^ *p* < 0.01 vs. SuHx.

Based on the network pharmacology and molecular docking results, we further explored the effects of *Plumula Nelumbinis* on hemodynamics and right heart function of SuHx-induced PH rats. Since the core compounds (neferine, liensinine, and isoliensinine) belong to alkaloids, we firstly evaluated the therapeutic effect of TAPN on PH rats *in vivo*. The construction of the PH rat model and the TAPN dosing schedule are shown in Figure 5A. Compared with the control group, the RVSP and Fulton index of SuHx rats were significantly increased, accompanied by a marked increase in right heart dysfunction, manifested as a significant decrease in PAAT, PAAT/PAET, TAPSE, and CO; these phenomena were markedly reversed by TAPN (Figures 5B–J). Interestingly, our results showed that TAPN had no significant effect on PAET and heart rate in rats (Figures 5G,K).

### Effects of TAPN on pulmonary vascular remodeling in SuHx-induced PH rats

**FIGURE 6:**
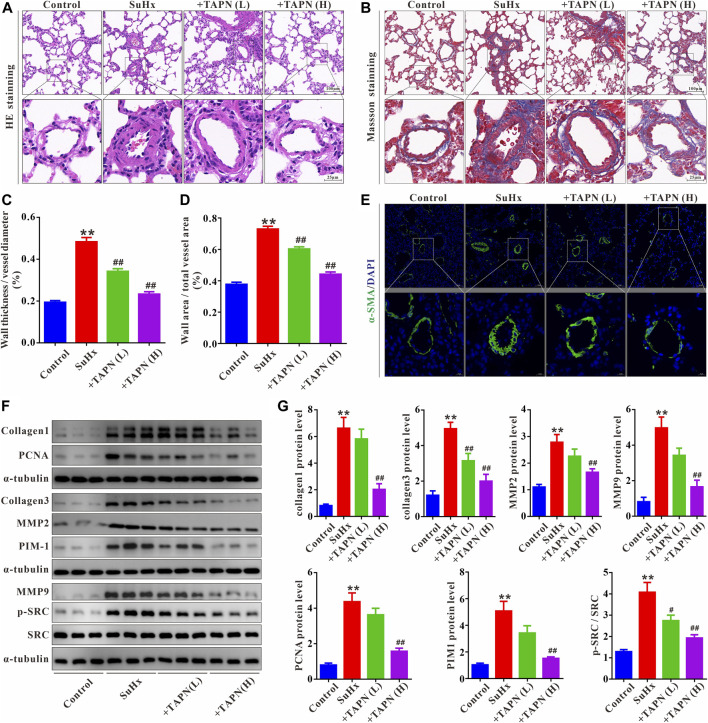
TAPN inhibits pulmonary vascular remodeling and collagen production in SuHx rats. **(A)** Representative images of HE staining **(B)** Representative images of Masson staining **(C)** The ratio of wall thickness to total vessel external diameter (WT%) **(D)** The ratio of wall area to total vessel area **(E)** Representative images of immunofluorescence staining (labeling of pulmonary vascular smooth muscle layer with α-SMA) **(F–G)** The protein expression of collagen1, collagen3, MMP2, MMP9, PCNA, PIM1, p-SRC, and SRC in the lung tissue of rats. n = 9, Data are expressed as mean ± SEM. SuHx: the Su5416 + hypoxia group; +TAPN (L): SuHx + TAPN low dose group (5 mg/kg/d); +TAPN (H): SuHx + TAPN high dose group (10 mg/kg/d); ^**^ *p* < 0.01 vs. Control; ^#^ *p* < 0.05, ^##^ *p* < 0.01 vs. SuHx.

Subsequently, we further evaluated the effect and underlying mechanism of TAPN on SuHx-induced pulmonary vascular remodeling in PH rats. HE and Masson staining showed that the pulmonary vascular remodeling and collagen production was significantly increased in SuHx-induced PH rats, manifesting as a significant increase in the ratio of WT% and WA% (Figures 6A–D). Immunofluorescence results showed that the pulmonary vascular smooth muscle layer (marked by α-SMA) was significantly thickened in SuHx rats (Figure 6E). In addition, western blot results showed that the protein expressions of collagen1, collagen3, MMP2, MMP9, and PCNA in the lung tissue of the SuHx group were significantly increased, accompanied by a significant increase in the expressions of PIM1 and p-SRC; these phenomena were markedly attenuated by TAPN (Figures 6F,G).

### Effect of *Plumula Nelumbinis* on hypoxia-induced proliferation and migration of PASMCs

**FIGURE 7:**
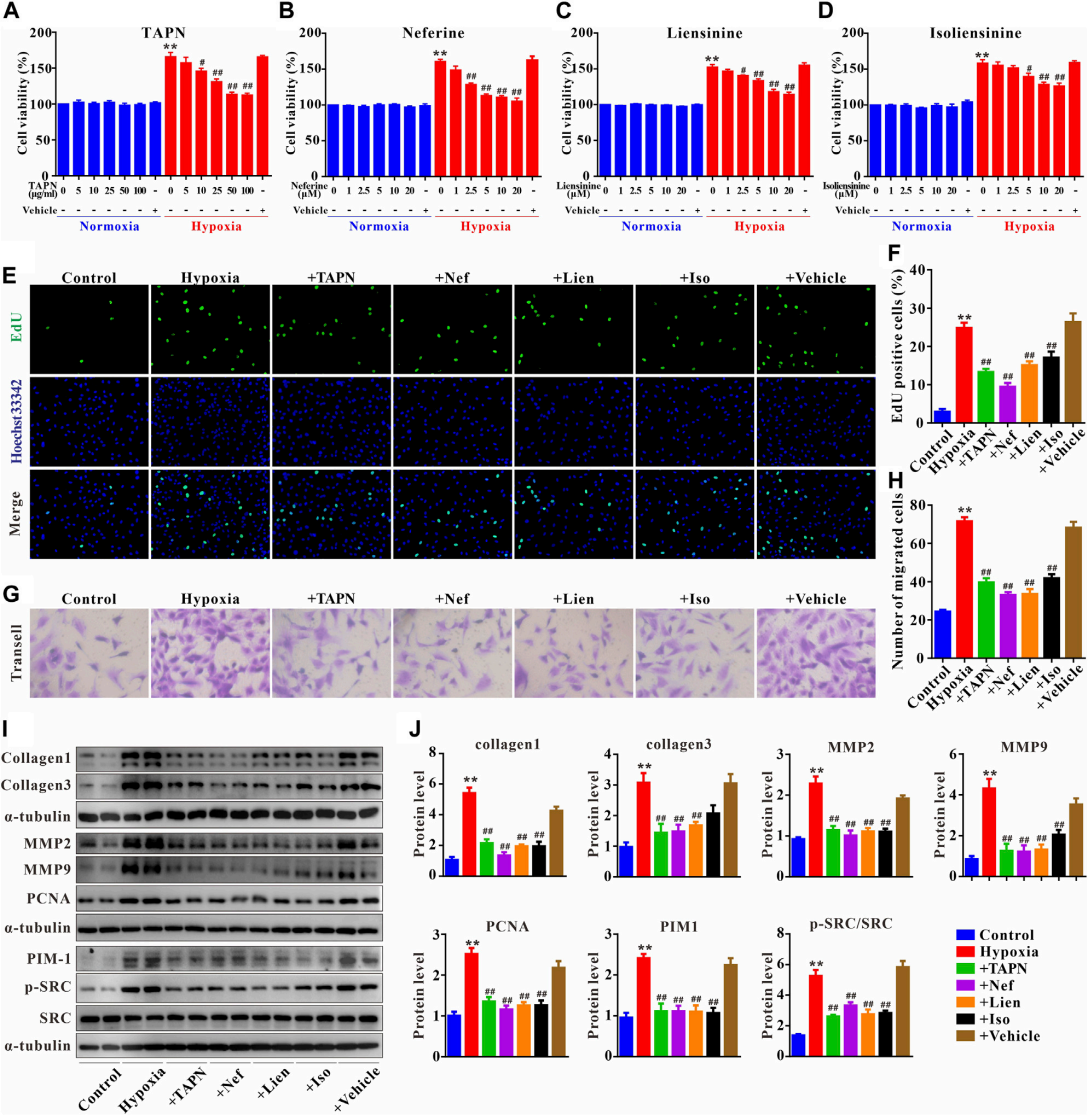
*Plumula Nelumbinis* attenuated hypoxia-induced proliferation and migration of PASMCs through inhibition of the activation of SRC or PIM1. **(A–D)** Effects of TAPN, neferine, liensinine, and isoliensinine on the proliferation of PASMCs induced by hypoxia (n = 3). **(E)** Representative images of EdU staining **(F)** Statistical analysis of EdU positive cells. **(G)** Representative images of crystal violet staining **(H)** Statistical analysis of cell migration **(I–J)** The protein expression of collagen1, collagen3, MMP2, MMP9, PCNA, PIM1, p-SRC, and SRC in PASMCs; n = 8–12, data are expressed as mean ± SEM. +TAPN: hypoxia + total alkaloids of *Plumula Nelumbinis* (50 μg/ml); +Nef: hypoxia + neferine (5 μM); +Lien: hypoxia + liensinine (10 μM); +Iso: hypoxia + isoliensinine (10 μM) ^**^ *p* < 0.01 vs. Control; ^#^ *p* < 0.05, ^##^ *p* < 0.01 vs. Hypoxia.

The primary PASMCs were cultured under hypoxia for 48 h to establish a cell proliferation and migration model. As shown in Figures 7A–D, under normoxic conditions, TAPN, neferine, liensinine, and isoliensinine did not affect the viability of PASMCs, suggesting that TAPN (5–100 μg/ml) and the 3 alkaloid active components (1–20 μM) have no toxic effects on PASMCs. However, under hypoxic conditions, the abnormal proliferation of PASMCs was inhibited by TAPN, neferine, liensinine, and isoliensinine in a dose-dependent manner. The drug concentration (TAPN: 50 μg/ml; neferine: 5 μM; liensinine and isoliensinine: 10 μM) for subsequent experiments was determined based on the results of CCK-8. Further results showed that hypoxia could promote the proliferation and migration of PASMCs (Figures 7E–H), along with an increase in the protein expressions of collagen1, collagen3, MMP2, MMP9, and PCNA in PASMCs (Figures 7I,J), these phenomena were markedly attenuated in the treatment of TAPN, neferine, liensinine, and isoliensinine.

Finally, we evaluated the underlying mechanisms of *Plumula Nelumbinis* on hypoxia-induced hyperproliferation and migration of PASMCs. Compared to the control group, the protein expression of p-SRC and PIM1 in the hypoxia group was significantly upregulated; these increases were attenuated by TAPN, neferine, liensinine, and isoliensinine (Figures 7I,J).

## Discussion

In this study, the anti-PH effect and potential mechanisms of the main active components of *Plumula Nelumbinis* were evaluated by network pharmacology, molecular docking, *in vivo*, and *in vitro* experimental verification. Firstly, we screened out the main active components of *Plumula Nelumbinis* against PH by using network pharmacology and molecular docking analysis. Then, by constructing a SuHx-induced PH rat model and a hypoxia-induced PASMCs proliferation model, we further revealed that the anti-PH mechanism of *Plumula Nelumbinis* might be achieved by inhibiting the proliferation and migration of PASMCs, and its mechanism is related to the inactivation of SRC and PIM1.

PH is a malignant cardiopulmonary vascular disease characterized by pulmonary vascular remodeling. With the continuous improvement of diagnosis and treatment technology in recent years, the 5-year survival rate of patients with PH has reached 53.6% (Gall et al., 2017). Unfortunately, PH is still an intractable disease for the current therapies can only alleviate patients’ symptoms and cannot effectively reverse the process of vascular remodeling. Therefore, it is an urgent clinical problem to seek more precise and effective interventions to prevent or treat PH. TCM has been used to treat PH through a multi-level and multi-target approach (Xue et al., 2021). The rise of network pharmacology, characterized by “multiple compounds-multiple targets-multiple signaling pathways”, provides an effective way to systematically explore the internal mechanism of TCM in treating PH (Nogales et al., 2021).

*Plumula Nelumbinis* is composed of various active ingredients, of which alkaloids are one of the main active ingredients. Recent studies showed that TAPN has the protective effect of inhibiting vascular remodeling in spontaneously hypertensive rats (Li Q et al., 2019). In addition, Jun et al. demonstrated that TAPN could inhibit the proliferation and migration of vascular smooth muscle cells, thereby inhibiting the carotid artery restenosis induced by balloon injury (Jun et al., 2016). The above studies have shown that TAPN plays a cardiovascular protective effect by regulating the proliferation and migration of vascular smooth muscle cells. However, its effect on PH is still unclear. In this study, we first predict the potential therapeutic targets of *Plumula Nelumbinis* against PH by using network pharmacology combined with molecular docking. Our prediction results indicated that the main active components of *Plumula Nelumbinis* in treating PH were alkaloids, including neferine, liensinine, and isoliensinine. Subsequently, we further prove that TAPN has a therapeutic effect on PH by using the SuHx-induced rat model, manifested as a significant decrease in RVSP, along with a significant improvement in cardiovascular remodeling and right heart function.

PH is a complex disease involving multiple targets and pathways. GO enrichment analysis showed that the anti-PH effect of *Plumula Nelumbinis* might be related to multiple biological processes and molecular functions, including regulation of MAPK cascade, wound healing, endopeptidase activity, and protein serine/threonine/tyrosine kinase activity. KEGG enrichment analysis revealed that its anti-PH effect might involve regulating cancer signaling pathways (hsa05205: proteoglycans in cancer; hsa05200: pathways in cancer; hsa05215: prostate cancer). It is worth noting that, comparable to cancer, PH is also a proliferative disease with high mortality and is thus known as the “cancer” of the cardiopulmonary vascular system. However, unlike cancer, which is caused by the excessive proliferation of cancer cells, PH is mainly caused by the excessive proliferation of vascular cells, especially the PASMCs in the media layer. Previous studies have demonstrated that neferine and isoliensinine have protective effects on angiotensin Ⅱ (Ang Ⅱ)-induced hyperproliferation of smooth muscle cells (Xiao et al., 2006; Li et al., 2010; Zheng et al., 2014). In the present study, we found that the alkaloid compounds in *Plumula Nelumbinis* (including TAPN, neferine, liensinine, and isoliensinine) have the ability to inhibit the abnormal proliferation and migration of PASMCs induced by hypoxia, suggesting that the therapeutic effect of *Plumula Nelumbinis* on PH vascular remodeling may be related to the inhibition of excessive proliferation and migration of PASMCs.

SRC, a proto-oncogene tyrosine-protein kinase, has been shown to mediate the occurrence and development of hypoxic PH by regulating vasoconstriction, cell proliferation, and apoptosis (Gao and Raj, 2020; Norton et al., 2020). Recently studies have proved that the effect of SRC in promoting hypoxic PH is closely related to the regulation of the activation of downstream proteins such as HIF-1α, HIF-2α, signal transducer and activator of transcription 3 (STAT3), and NADPH oxidase (Gao and Raj, 2020). In addition, inhibition of SRC activation by drug intervention could effectively reverse vascular remodeling in experimental PH (Pullamsetti et al., 2012), further clarifying the vital role of SRC in the promotion of PH. PIM1, a serine/threonine family kinase, has now been recognized as a novel biomarker and therapeutic target in the development of PH (Satoh et al., 2020). A large number of studies have demonstrated that PIM1 is overactivated in patients with PH, preclinical animal models, or cell models (Paulin et al., 2011; Ge et al., 2021), and inhibition of PIM1 expression by drug intervention could also effectively delay the progression of PH (Lampron et al., 2020). NOS3, also known as eNOS, is mainly expressed in endothelial cells and could play a protective role in PH vascular remodeling by regulating nitric oxide synthesis (Hua et al., 2018). The above studies show that SRC and PIM1 may participate in the development of PH by promoting the proliferation of PASMCs. On the contrary, NOS3 may alleviate the PH process by regulating the function of endothelial cells. In this study, we found that the protein expressions of p-SRC and PIM1 in lung tissues were significantly increased in SuHx-induced PH rats, and these increases were suppressed in the presence of TAPN. Expectedly, our *in vitro* experiment results further showed that the expression of PIM1 and p-SRC in PASMCs were significantly increased under hypoxic conditions; these phenomena were attenuated in the presence of TAPN, neferine, liensinine, and isoliensinine. These findings are consistent with our network pharmacology and molecular docking predictions, suggesting that the alkaloids of *Plumula Nelumbinis* may inhibit the proliferation and migration of PASMCs by targeting SRC and PIM1 thereby achieving the purpose of treating PH. It is worth noting that we also detected the expression of eNOS in PASMCs after hypoxia treatment. However, the results showed that the basal expression of eNOS in PASMCs was deficient and challenging to detect. Further studies are needed to confirm the role of *Plumula Nelumbinis* on eNOS activity in pulmonary artery endothelial cells.

## Conclusion

**FIGURE 8:**
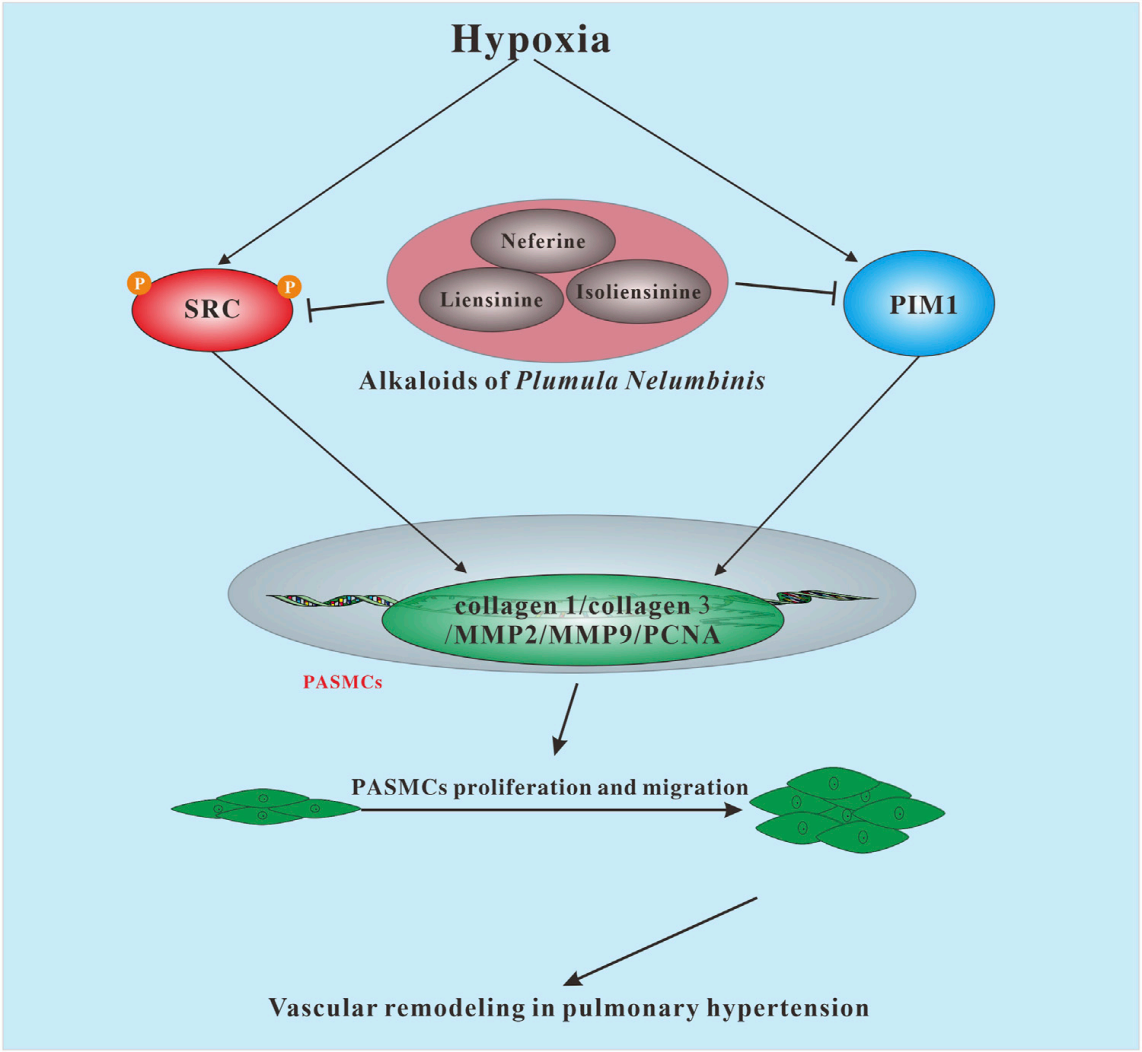
The possible mechanism of *Plumula Nelumbinis* against pulmonary hypertension.

In summary, this study demonstrated for the first time that TAPN (such as neferine, liensinine, and isoliensinine) exerts a therapeutic effect on PH by inhibiting the excessive proliferation and migration of PASMCs, and its mechanism is related to promoting the inactivation of SRC and PIM1 (Figure 8). The above results can provide an experimental basis for the development of new drugs for PH.

## Data Availability

The datasets presented in this study can be found in online repositories. The names of the repository/repositories and accession number(s) can be found in the article/supplementary material.
